# Mitochondrial autophagy: molecular mechanisms and implications for cardiovascular disease

**DOI:** 10.1038/s41419-022-04906-6

**Published:** 2022-05-09

**Authors:** Anqi Li, Meng Gao, Bilin Liu, Yuan Qin, Lei chen, Hanyu Liu, Huayan Wu, Guohua Gong

**Affiliations:** 1 Institute for Regenerative Medicine, Shanghai East Hospital, School of Life Sciences and Technology, Tongji University, Shanghai, 200092 China; 2 Department of Pharmacy, Shanghai East Hospital, Tongji University, Shanghai, 200120 China

**Keywords:** Mitochondria, Cardiovascular diseases

## Abstract

Mitochondria are highly dynamic organelles that participate in ATP generation and involve calcium homeostasis, oxidative stress response, and apoptosis. Dysfunctional or damaged mitochondria could cause serious consequences even lead to cell death. Therefore, maintaining the homeostasis of mitochondria is critical for cellular functions. Mitophagy is a process of selectively degrading damaged mitochondria under mitochondrial toxicity conditions, which plays an essential role in mitochondrial quality control. The abnormal mitophagy that aggravates mitochondrial dysfunction is closely related to the pathogenesis of many diseases. As the myocardium is a highly oxidative metabolic tissue, mitochondria play a central role in maintaining optimal performance of the heart. Dysfunctional mitochondria accumulation is involved in the pathophysiology of cardiovascular diseases, such as myocardial infarction, cardiomyopathy and heart failure. This review discusses the most recent progress on mitophagy and its role in cardiovascular disease.

## Facts

Several distinguished molecular pathways mediate mitophagy.

Mitophagy selectively degrades damaged mitochondria is essential for mitochondrial homeostasis.

The mitochondrial fragment is generally a prerequisite for mitophagy.

Mitophagy involves various cardiovascular diseases, including atherosclerosis, ischemia-reperfusion injury, cardiomyopathy, hypertrophy, and heart failure.

## Open Questions

What is the relationship between mitophagy and cell autophagy?

How to control the optimal regulation of mitophagy?

How to restore the homeostasis of dysfunctional mitochondria?

## Introduction

Cellular homeostasis is a prerequisite for the optimal functional performance of cells [1]. Autophagy, as a conventional regulation mechanism of the homeostasis of eukaryotic cells, is highly conserved throughout the evolutionary process. It is responsible for removing cellular components such as accumulated proteins and damaged organelles in cells to maintain intracellular homeostasis [1, 2]. As a degradation pathway, autophagy balances the biosynthesis and catabolism of macromolecules to protect organisms against diverse pathologies, including cancer, aging, neurodegeneration, and heart disease [1, 3–5].

Mitochondria, as the most important energetic cellular organelles, play a pivotal role in intracellular homeostasis [6]. Maintenance of mitochondrial function and integrity is crucial for normal cell physiology [7]. As an energy-rich compound, adenosine triphosphate (ATP) is mainly produced by mitochondria. In addition, mitochondria are also involved in modulating second messenger levels, such as calcium ions (Ca^2+^), cAMP and reactive oxygen species (ROS) [8]. It has been proved that dysfunctional mitochondria not only in response to decrease ATP production but also increase oxidative stress [9, 10]. Dysfunctional mitochondria can also perturb calcium homeostasis due to the closed contact between mitochondria and the endoplasmic reticulum. The Ca^2+^ signal converted from physiological into a pathological is concerned as a pathological marker [11]. Thus, mitochondrial quality needs to be well controlled in cells. Nuclear and mitochondrial DNA jointly regulate mitochondria, which makes them sensitive to environmental stimulation. The different contexts of mutations in mtDNA or nuclear DNA will result in different clinical phenotypes of disease [12]. Damaged mitochondria need to be selectively removed through mitochondrial autophagy (mitophagy), the process of which is controlled by nuclear coding proteins [13].

Mitophagy and mitochondrial biogenesis are two opposite processes in determining the number of mitochondria, both of which also are key regulators of mitochondrial quality and steady-state mitochondrial turnover [14]. Mitophagy as targeting degrade severely damaged mitochondria plays a more important role. It has been known that reactive oxygen species (ROS) are generated from the electron transport chain, which challenges mitochondrial structure and function [8]. If damaged mitochondria are not eliminated in time, they would contaminate the healthy mitochondria through reactive oxygen species (ROS)-induced ROS release (RIRR) [15]. RRIR is a vicious downward spiral, amplifying the ROS signal, thus eventually causing irreversible damage to cells. Excessive ROS impacts cellular proliferation and triggers the peroxidation of lipids, impairment of DNA and apoptosis [8]. Mitophagy, as a selective autophagy pathway to mediate the clearance of damaged or senescent mitochondria, is critical for maintaining cellular function [16]. Abnormal mitophagy is closely related to various diseases, particularly in organisms with high energy demand heart, including heart failure, hypertrophy and ischemia-reperfusion [17].

In this review, we focus on the current understanding of mitophagy machinery and how mitophagy is regulated in cardiovascular disease.

### Mitophagy

Mitochondria, as the powerhouse, play a pivotal role in cells. Damaged mitochondria need to be precisely removed timely to keep proper mitochondrial functions. Selectively removal or degradation of damaged mitochondria by autophagy are termed mitophagy [17, 18]. As a double-membrane organelle, Mitochondria are mainly responsible for intracellular aerobic respiration and producing energy in oxidative phosphorylation. The normal oxidative phosphorylated process will generate adenosine triphosphate (ATP) and reactive oxygen species (ROS) [19]. Not only do mitochondria play a vital role in maintaining cell homeostasis and regulating cell proliferation, but they are also involved in many cell activities, such as calcium signal transduction [20], metabolic synthesis, programmed death [21], and tumorigenesis [22, 23].

As a defense mechanism, mitophagy can selectively remove damaged and dysfunctional mitochondria in cells to maintain the quality of mitochondria, thereby keeping mitochondrial physiological functions. In a word, mitophagy is indispensable for cells to clear abnormal mitochondria in response to stress. Mitochondrial damage is usually associated with programmed cell death, inflammation and aging. Increased or accumulated damaged mitochondria will aggravate the occurrence and the pathogenesis of many diseases [9]. Under normal physiological conditions, the basal level of mitophagy in cells can make dysfunctional mitochondria timely recognized and removed, thereby providing sufficient raw materials for fresh mitochondria and ensuring the energy supply of cells to preserve cellular homeostasis. In turn, insufficient mitophagy will lead to the accumulation of dysfunctional mitochondria. Consequently, intracellular ATP level decreases, and ROS level will elevate. Excess mitochondrial ROS reacts with proteins, lipids, and nucleic acids, causing oxidative damage and apoptosis. Impaired mitophagy can lead to various diseases, including cancer, neurodegenerative diseases, and cardiovascular diseases. Increasing mitophagy in response to different stress conditions can preserve mitochondrial quality by maintaining cellular ATP level, reducing the oxidative damage caused by ROS, and selectively removing the damaged mitochondria in the cell. Healthy mitochondrial quality control is crucial for the metabolism of cells.

In recent studies, it has been demonstrated that accelerating cellular metabolism can prevent the development of many metabolic diseases. However, too much water drowned the miller. Excessive clearance of mitochondria leads to loss of mitochondria and increases oxidative species such as ROS, which is deleterious in terms of normal cellular requirements. Therefore, precise and proper regulation of mitophagy is helpful for cells to keep homeostasis.

### Pathways of mitophagy

Mitophagy can be divided into ubiquitin-mediated mitophagy and receptor-mediated mitophagy. The ubiquitin-mediated mitophagy includes PINK/Parkin, and other ubiquitin-mediated pathway. The receptor-mediated mitophagy pathways include BNIP3 (Bcl-2 and adenovirus E1B19 kDa-interacting protein 3) mediated mitophagy, FUNDC1-mediated mitophagy, and lipid-mediated mitophagy.

### PINK1/Parkin-mediated mitophagy

**Fig. 1:**
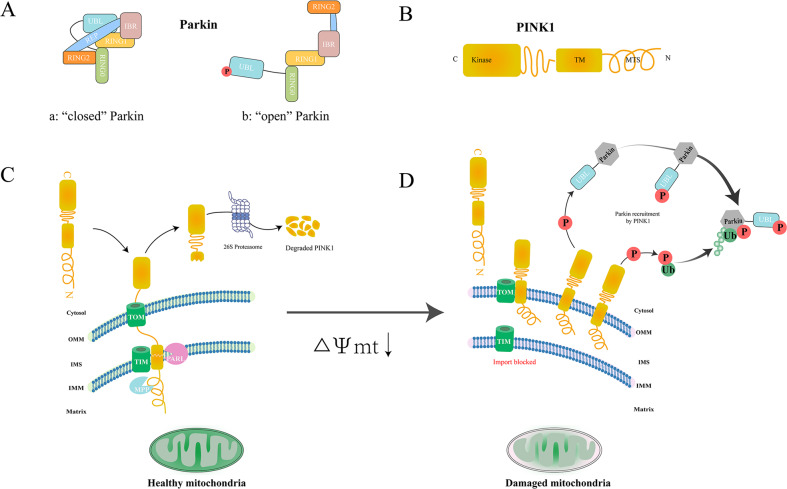
PINK1/Parkin-mediated mitophagy. **A** Under normal conditions, the conformation of Parkin is closed, which contains an N-terminal ubiquitin-like (UBL) domain, repressor element of Parkin (REP), four zinc-coordinating RING-like domains: RING0, RING1 (H302 to R305 motif), in-between RING (IBR), and RING2. Once Parkin is activated, its conformation becomes open and the UBL domain is released. **B** Domain features of PINK1: PINK1 possesses a Ser/Thr kinase domain (Kinase) close to C-terminal, followed by an α-helical transmembrane (TM) segment, and N-terminal mitochondrial targeting sequence (MTS). **C** PINK1 is continuously imported into healthy mitochondria then degraded. **D** Positive cycles for PINK1 and Parkin Recruitment on damaged mitochondria.

In 1998, PRKN was discovered as the causative gene of autosomal recessive juvenile parkinsonism (AR-JP) [24]. Parkin protein is composed of 465 amino acids, including ubiquitin-like domain (UBL), repressor element of Parkin (REP), four zinc-coordinating RING-like domains: RING0, RING1 (H302 to R305 motif), in-between RING (IBR), and RING2 [25] (Fig. 1A). As an E3 ubiquitin ligase, Parkin mainly consists of three families: homologous to the C-terminus of E6-AP (HECT ligase) can react with ubiquitin to form thioester-linked intermediates. Really Interesting New Gene (RING) ligase can transfer ubiquitin from the E2 ubiquitin-conjugating enzyme onto the substrate by binding E2 enzyme. RING-Between-RING (RBR) ligase is referred to as a RING-HECT hybrid because it combines the characteristics of RING-type ligases and HECT-type ligases. Parkin is a member of RBR E3 ubiquitin ligases that can modulate ubiquitination of various proteins in cytosolic and outer mitochondrial membrane. Parkin is closely related to mitochondrial morphology, mitochondrial dynamics, mitochondrial quality control, autophagy and other physiological activities in cells. Under normal conditions, Parkin can maintain its structural stability and inhibit its ubiquitin ligase activity due to its closed conformation. In “closed” Parkin, C431 site, the catalytic center of RING2 region is blocked, and the E2 ubiquitin-conjugating enzyme binding site in the RING1 region is also blocked; both of which can inhibit ubiquitin–thioester formation. In the PINK1/Parkin mitochondrial autophagy pathway, Parkin acts as a downstream protein of PINK1 [25, 26].

In 2004, PINK1 is also reported as another pathogenic gene of Parkinson’s disease. Similar to PRKN, PINK1 is inherited in an autosomal recessive manner [27]. PINK1 is a serine/threonine-protein kinase composed of 581 amino acids. It is mainly involved in oxidative stress in cells, releasing transmitters, autophagy, apoptosis and other physiological activities. As a mitochondrial kinase, PINK1 has been discovered as the primary detector of mitochondrial damage. The N-terminal domain of PINK1 is regarded as a mitochondrial target signal (MTS), which is followed by a hydrophobic transmembrane domain (TM) as a terminal transfer signal of the mitochondrial inner membrane. Resides 156 to 509 constitutes a serine/threonine kinase domain, and to the end followed by C-terminal domain that serves as a retention signal or others for the outer mitochondrial membrane (OMM). (Fig. 1B)

Physiological PINK1 protein is continuously imported into healthy mitochondria through MTS and the mitochondrial membrane potential (ΔΨm) [28, 29]. Subsequently, the N-terminal MTS of PINK1 was cleaved by matrix processing peptidase (MPP) including MPPα and MPPβ while MTS reaching matrix. Hydrophobic transmembrane helix within TM was cleaved by (PINK1/PGAM5-associated rhomboid-like protease) PARL, the catalytic ability of which is indispensable for Parkin recruitment in mitophagy [30]. After this series processing, a 64 kDa full-length forms of PINK became 52 kDa shorter forms, which are released into the cytoplasm. The cytoplasmic PINK1 is rapidly degraded by the Ubiquitin-Proteasome system (UPS) via the N end–rule pathway. In damaged depolarized mitochondrial, PINK1 cannot be transported to IMM to be cleaved, causing the accumulation of PINK1 on OMM to form dimers. PINK1 dimers are activated by phosphorylation at S228 and S402 [31], which is required for recruiting Parkin to OMM to initiate mitophagy. Ser65-phosphorylated ubiquitin (pUb) on PINK1 interacts with RING0, RING1, and IBR regions of Parkin, which contributes to straightening a helix in the RING1 domain. Such conformational changes in “opened” Parkin cause the UBL domain to be released from its core structure (Fig. 1A, B) [32, 33]. Subsequently, PINK1 phosphorylates Parkin on its UBL domain at S65, which promotes the interaction between UBL and RING0 to release the catalytic RING2 domain that is mutually inhibited with RING0, thereby further activating E3 ubiquitin ligase activity of Parkin [34, 35]. The subsequent Parkin activation and ubiquitination proteins of OMM would provide extra substrates for PINK1, recruits more Parkin. Such a positive cycle amplifies mitophagy signals [36] (Fig. 1C, D).

Mitochondrial fusion protein 2 (Mfn2) is localized on OMM and phosphorylated at S442 and T111 by PINK1. The phosphorylated Mfn2 act as a Parkin ubiquitination substrate to prevent impaired mitochondria fusing with the healthy organelle [37]. Increasing evidence implicates that the fusion machinery components on damaged mitochondria were degraded by UPS. Parkin will furtherly utilizes UPS to promote the division of damaged mitochondria. The Miro GTPases also are substrates of Parkin, and the damaged mitochondria were segregated from the mitochondrial network by Miro to form an insulating membrane, which is necessary for subsequent mitophagy.

The formation of isolation membrane by Lc3 largely depend on two kinds of ubiquitin-like reaction. The carboxyl-terminal region of pro-Lc3 form is cleaved by cysteine protease Atg4 to form Lc3-I by exposing its glycine residues. The Lc3-II formation is dependent on the binding PE to Lc3- I by Atg7 (an E1-like enzyme) and Atg3 (a specific E2-like ligase). The second ubiquitin-like reaction is driven by Atg7 and Atg10 (an E2-like enzyme) to combine Atg12 with Atg5 to form Atg12~5 conjugates, which subsequently bind to Atg16 dimer to form Atg12~5/16 complex. Finally, the Atg12~5/16 complex promotes Atg8 bind with (PE) on the expanding autophagosomal membrane via its E3-like activity. It is thought that the isolation membranes recognize the damaged mitochondria via the LIR motif (Lc3-interacting region), which will wrap the mitochondria to form autophagosomes [38, 39].

The process of linking isolation membrane and damaged mitochondria is mediated by autophagic adaptor proteins, including sequestosome1 (P62 /SQSTM1), a neighbor of Brca1 gene (NBR1), Nuclear dot protein 52 kDa (NDP52), Optineurin (Optn), Tax1-binding protein 1(TAX1BP1) [40]. OMM proteins degradation is induced by K63-linked ubiquitin that recruits mitochondrial adaptor proteins and interacts with Lc3 anchored to the autophagosome membrane. It has been claimed that p62 preferentially locates between adjacent mitochondria and promotes the aggregation of damaged mitochondria through polymerization [41, 42]. But other scientists argued that OPTN and NDP52 are the main adaptors in PINK1/ Parkin-mediated mitophagy, rather than P62 and NBR1 [5, 43, 44]. NDP52 and OPTN are mainly located in the OMM of damaged mitochondria. TBK1 is activated and anchored to p62 via phosphorylation at S172 by OPTN. TBK1 phosphorylates P62 at S403 and OPTN at S177 to promote their binding with UB chains or Lc3. OPTN and NDP52 then initiate the autophagy process by recruiting autophagy-related unc-51-like autophagy-activating kinase1 (ULK1), Double FYVE-containing protein 1(DFCP1) and WD repeat domain phosphoinositide-interacting protein 1(wipi-1). AMBRA1 as a Parkin interactor can localize to OMM to enhance mitochondrial clearance via binding to Lc3 through its LIR motif [45]. In addition, AMBRA1 also function despite of Parkin and p62 [46]. TBC1D15/17 (TBC1 domain family member 15/17) also assists in elongating the phagophore membrane, both of which can interact with OMM-anchored mitochondrial fission protein 1 (Fis1) [47–49].

**Fig. 2:**
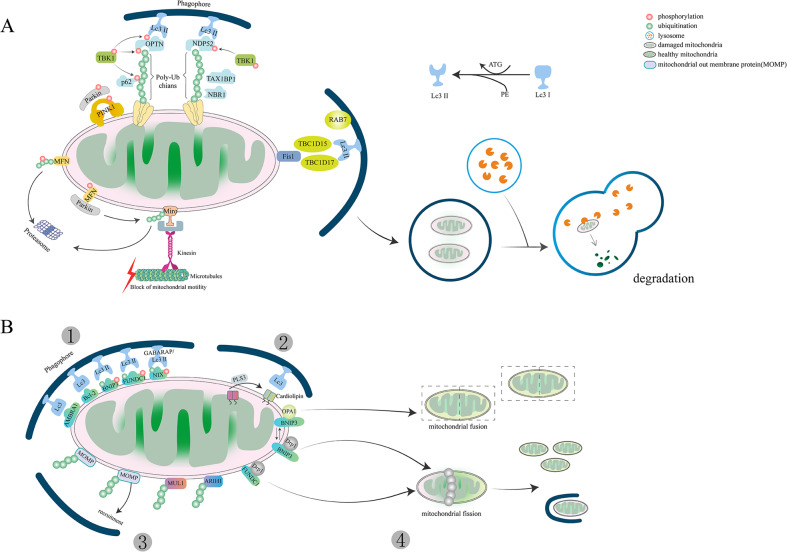
Pathways of mitophagy. **A** Parkin-dependent mitophagy: While mitochondria became damaged (pink), PINK1 is accumulated on the OMM, promoting Parkin recruitment to ubiquitinate several outer membrane components. Phosphorylated Poly-Ub chains on mitochondrial proteins modify other proteins and serve as an ‘eat me’ signal for the autophagic machinery. TBK1 interacts with Ub chains by phosphorylating OPTN, which promotes mitochondrial clearance. The PINK1-Parkin pathway modulates mitochondrial dynamics and inhibits damaged mitochondria entering the mitochondrial network by targeting Mfn and Miro for proteasomal degradation. **B** Other pathways of Parkin-independent mitophagy: (1) Bnip3, Bnip3L/Nix, FUNDC1 and other receptor-mediated mitophagy; (2) Lipid-mediated mitophagy; (3) E3 ubiquitin ligases or ubiquitin-mediated mitophagy; (4) Mitophagy and mitochondrial dynamics. Mitophagy receptors such as BNIP3 and FUNDC1 promote the fission of damaged organelles through the disassembly and release of OPA1 and the recruitment of DRP1.

Finally, the damaged or senescent mitochondria are enclosed into autophagosomes and then delivered to lysosomes for degradation via fusing with lysosomes to form autolysosomes. Mitophagy is a highly conserved process, which also requires traffic GTPase Rab7 to regulate lysosomal transport [47, 50]. PINK1 and Parkin are degraded after the lysosome-dependent degradation process (Fig. 2A).

### Other ubiquitin-mediated mitophagy

Mitochondrial ubiquitination plays a central role in mitophagy. Apart from Parkin, other E3 ubiquitin ligases are also involved in the clearance of damaged mitochondria. Mitochondrial ubiquitin ligase activator of mitochondrial E3 ubiquitin-protein ligase 1 (MUL1), an E3 ubiquitin ligase, contains two TM domains anchored to OMM and contains a RING finger (RNF) domain facing the cytoplasm [51]. It also has multiple common mitochondrial substrates with Parkin, including Drp1, Mfn1, and Mfn2. Studies on Drosophila and mice have shown that MUL1 is parallel to PINK1/ Parkin-mediated mitophagy and can compensate for the deletion of PINK1/ Parkin to rescue its phenotype [51]. Meanwhile, studies in HeLa cells have suggested that MUL1 can directly interact with GABARAP protein via the LIR-like motifs in the RING finger domain [52].

MUL1 not only plays a ubiquitination role as E3 ubiquitin ligase but also directly participates in mitophagy as a mitochondrial receptor. ULK1 is a newly discovered substrate of MUL1. The MUL1-regulated mitophagy process is independent of Parkin and FUNDC1, but needs ATG5 and ULK1 [53]. There is growing evidence revealed that mitophagy can still proceed without Parkin [40]. It is widely believed that the main regulators of mitophagy are tumor suppressors. Still, the presence of RBR E3 ubiquitin-protein ligase 1 (ARIH1) challenges this view because it has been shown to protect cancer cells from chemotherapy-induced death [54]. ARIH1 is mainly expressed in pluripotent stem cells and various cancer cells, especially lung cancer cells [55]. It belongs to the RBR E3 ubiquitin ligase, which shows many similar structures and substrates with Parkin. Both Parkin and ARIH1 act with the ubiquitin-conjugating enzyme UBCH7 (UBE2L3), but they lack intrinsic E3-independent reactivity with lysine. Parkin substrates including Mfn2, NPD52 or OPTN are not required in ARIH1-mediated mitophagy, suggesting that ARIH1 can target a group of mitochondrial proteins different from Parkin substrates to induce mitophagy (Fig. 2B). Mitochondrial-derived vesicle (MDV) is discrete vesicles from OMM or IMM, which are targeted to peroxisomes, lysosomes, endosomes, and phagosomes. MDV buds off from mitochondria and incorporates specific mitochondrial cargo especially oxidized cargo to the late endosome. So far, MDV has only been found in some cell types, such as hepatic cells and cardiac cells. An increased number of MDVs can be found in H9C2 cardiac myoblasts in response to stress [56]. MDVs in the healthy heart and acute stress heart can trigger mitophagy. MDVs are involved in the clearance of mildly damaged mitochondria, and its formation depends on PINK1 and Parkin but is independent of Drp1 [57]. Whether MDVs are present in every cell type remains to be explored.

### BNIP3 and BNIP3L/Nix-mediated mitophagy

Bcl-2 and adenovirus E1B 19-kDa-interacting protein 3 (BNIP3) and BNIP3-like (BNIP3L, also called Nix) are homologous members of Bcl-2 family proteins [58]. They are constitutively expressed on the MOM and are known as an apoptotic protein at first. Both play a role as mitophagy receptors to regulate PRKN/PARK2-independent mitophagy. BNIP3L has two LIR domains, a minimal essential region (MER), a TM domain, a BCL2 homolog 3 domain (BH3) inducing apoptosis. The C-terminal LIR domain was considered as an essential component to initiate mitophagy and interact with Lc3 due to phosphorylation. Recent studies in HeLa cells demonstrate that BNIP3L/Nix can increase its interaction with Lc3/GABARAP by phosphorylation at S34 and 35 adjacent to the LIR motif [59]. In particular, phosphorylation of the LIR domain can promote mitophagy and deletion of LIR has a reverse result. However, some scientists argued that is MER domain rather than LIR domain closed to the C-terminus is essential for BNIP3L -mediated mitophagy [60]. The deletion of the MER domain in BNIP3L failed to induce mitophagy. TM domain facilitates BNIP3L OMM location and promotes BNIP3L homodimerization through phosphorylation, which is essential for mitophagic activity [13]. Currently, the understanding of BNIP3L regulated mitophagy remains limited. BNIP3L helps BECN release from the Bcl-BECN complex, which promotes autophagosome formation. Besides, BNIP3 and BNIP3L /Nix can bind to the Rheb proteins to inhibit mTOR activation through their N-terminal and enhance mitophagy. BNIP3L relies on LIR to directly interact with the Atg8 family, such as Lc3, after phosphorylation and ubiquitination under hypoxia (Fig. 2B) [61]. When BNIP3L is accumulated to a certain level on OMM, it will lead to mitochondrial membrane potential loss and thus initiate mitophagy [62, 63], but this causal relationship has been questioned. Some scientists argued that different from Parkin, the translocation of BNIP3 is independent of membrane potential loss. It has been found that the membrane potential still decreased when programmed mitophagy occurred in cardiac Progenitor cells (CPS) lacking BNIP3 [64]. Other scientists demonstrated that hypoxia does not seem to be the only condition that triggers BNIP3L-mediated mitophagy. In hypoxic colon carcinoma, hypoxia-induced mitophagy is independent of BNIP3L but activated by AMPK [65]. The relationship between hypoxia and BNIP3-mediated mitophagy needs further research. The mechanism of BNIP3L-mediated mitophagy is much controversial and needs to be explored in more detail for well understood. What’s more, specific diseases caused by BNIP3L mutations have also been rarely reported.

### FUNDC1-mediated mitophagy

FUNDC1 (FUN14 domain-containing protein 1) is another widely studied mitophagy receptor localized to OMM, which was first proposed in 2012 by Chen’s lab [66]. It contains three transmembrane domains consisting of three conserved α-helical stretches, a LIR motif closed to N-terminus in the mitochondrial cytoplasmic face and a C-terminus in the intermembrane space. Similar to BNIP3 and BNIP3L/Nix, FUNDC1 also interacts with Lc3-II through phosphorylation and dephosphorylation of LIR. FUNDC1 is dependent on hypoxia-induced dephosphorylation to promote mitophagy. In contrast, other proteins containing LIR motifs need phosphorylation to increase Lc3 binding affinity. What’ more, FUNDC1 also can interact with other Lc3 paralogues such as GABARAP, but the binding affinity between them is not as high as with Lc3-II. Under normal conditions, LIR activity of FUNDC1 is inhibited due to phosphorylation at tyrosine 18 (Tyr18) and Ser13 by Src kinase and Casein kinase 2 (CK2 kinase), respectively [66, 67]. Phosphoglycerate mutase family Member 5 (PGAM5) can dephosphorylate FUNDC1 at Ser13 under hypoxia or mitochondrial uncoupling. It has been suggested that phosphorylation of Tyr18 played a key role and phosphorylation of Ser13 plays an auxiliary role in FUNDC1-mediated mitophagy [68]. Phosphorylation of Tyr18 significantly reduced the binding affinity of LIR to Lc3, but phosphorylation of Ser13 had only a slight effect. UNC-51, like autophagy-activating kinase 1(ULK1), can also phosphorylate FUNDC1 at Ser17 on the LIR motif in HeLa cells and promote the interaction between FUNDC1 and Lc3 [69]. Intriguingly, Ser17 phosphorylation has a stimulative effect compared with Ser13 and Tyr18 phosphorylation on mitophagy in response to hypoxia or FCCP treatment. Unlike Parkin, ULK1 migrates to damaged mitochondria in response to hypoxia. The substrates of ULK1 and subcellular location remain unknown and need more research.

On the other hand, the E3 ubiquitin-ligase membrane-associated RING finger protein 5 (MARCH5) has been found as a supervisor to avoid excessive and inappropriate clearance of mitochondria by FUNDC1 degradation, which is a new substrate for MARCH5 [70]. Similar to Parkin, both of them were able to ubiquitinate OMM proteins. Nevertheless, the purpose of Parkin is recruit p62 and the isolation membrane, while MARCH5 specifically targets FUNDC1 for degradation to fine-tune mitophagy. It has been reported that MARCH5 ubiquitinated Lys119 of FUNDC1 for proteasome degradation, which was independent of FUNDC1 phosphorylation. In addition, MARCH5 knockdown significantly inhibited FUNDC1 degradation and enhanced mitophagy signals [71]. It has also been suggested that FUNDC1 regulates mitochondrial fusion and fission by interacting with optic atrophy protein 1(OPA1) and Drp1, which is a critical quality control event upstream of mitophagy (Fig. 2B) [72]. Other mitochondrial fusion-related protein such as Mfn1, Mfn2, and fission-related protein Fis1, MiD49 can also be ubiquitylated by MARCH5 [73]. These results imply that the role of E3 ubiquitin ligase is also varied in mitophagy.

### Lipid-mediated mitophagy

Some OMM-localized lipids, such as ceramide and cardiolipin, induce mitophagy via their LIR motif. Ceramide is a bioactive sphingolipid synthesized by ceramide synthase (CerS), including six isoforms (CerS1- CerS 6). The production of ceramides in the hepatic mitochondria relies on mitochondrial thioesterase and neutral ceramidase (NCDase) [74]. Under ER stress, ceramides are translocated from ER to OMM in preparation for mitophagy [75]. When they accumulate in cells to a certain extent, accompanied by accumulation of Beclin-1 and inhibition of Akt phosphorylation, cell death will happen. Ceramides located on the OMM interact with the ceramide-binding domain of Lc3-II to selectively remove damaged mitochondria. It has been reported that Lc3-II lipidation is necessary for ceramide binding during the mitophagy process [76]. C18-ceramides are preferentially synthesized from 18 carbon (C18) fatty acids catalyzed by CerS1. Overexpression of CerS1 or adding exogenous C18-ceramide can enhance ceramide-mediated mitophagy depending on Drp1 [77]. Drp1 determines OMM localization of ceramide to target autophagolysosomes. Drp1 knockdown disrupts ceramide localization in OMM and autophagolysosomes recruitment in tumor cells [78]. Mitochondrial fission is essential for ceramide-induced mitophagy due to Drp1 being the crucial regulator of mitochondrial fission.

Moreover, ceramides, along with activated BAX, form a ceramide channel in the phospholipid bilayer to release cytochrome c to activate apoptosis. Cardiolipin, as a negatively charged phospholipid similar to ceramide, is synthesized by cardiolipin synthase (CRD1). It is synthesized by mitochondria and makes up about 25 mol% of mitochondrial membrane lipids, which is also important in mitochondrial cristae formation and other mitochondrial functions and mitochondrial fusion [79]. The distribution of cardiolipin is highly asymmetric between IMM and OMM in healthy mitochondria, most of which about 96.5 mol% are confined to the IMM [80]. The cardiolipin binding site in Lc3 is believed to be at the N-terminal α-helices through computational modeling. Under normal conditions, cardiolipin locates at IMM and interacts with OPA1 to promote IMM protein fusion and regulate mitochondrial networks. Cardiac phospholipid cardiolipin is transferred to OMM via phospholipid scramblase-3 (PLS3) to bind to Lc3-II under stress (Fig. 2B) [81].

PLS3 is a newly recognized protein is responsible for phospholipid translocation between bilayer structures. Overexpressing PLS3 in HEK293 cells results in cardiolipin accumulation in OMM and enhanced intracellular ATP and mitochondrial respiration [81]. Nucleoside diphosphate kinase D (NDPK-D) is a hexameric intermembrane space protein that also assists cardiolipin-translocating. Knockdown endogenous NDPK-D reduced cardiolipin on OMM inducef by CCCP in Hela cells **(**Fig. 2B) [80].

### Mitophagy and mitochondrial dynamics

**Fig. 3:**
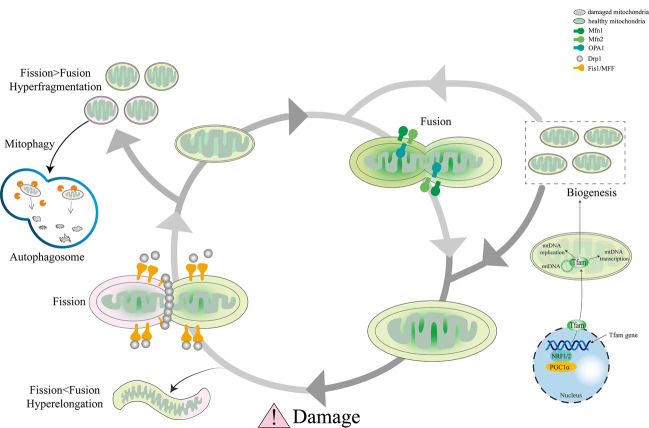
Consequences of mitochondrial dynamics. Damaged or senescent mitochondria divide into one healthy organelle that fuses with other healthy mitochondria to regenerate the mitochondrial pool and one severely depolarized organelle that is timely eliminated by autophagosomal engulfment. Thereby new mitochondria are generated by biogenesis to replace degraded mitochondria parts. The balance offset between mitochondrial fusion and fission leads to either hyperelongated or hyper fragmented mitochondria.

Mitochondria are highly active organelles undergoing continuous movement, fission, and fusion activity and response to environmental stimuli. Before being selectively eliminated by mitopahgy, a damaged mitochondrial will be asymmetrically divided into a healthy daughter and an impaired organelle via fission proteins. The mitochondrial fission proteins are constitutive of Drp1, FIS1, MFF proteins. The impaired organelle is degraded via mitophagy. The healthy daughter can fuse with other mitochondria to enable content mixing and maintain mitochondrial fusion proteins’ genetic integrity. Severely damaged mitochondria will be replaced by fresh mitochondria generated by mitochondrial biogenesis. Mitochondrial fusion consists of the outer membrane fusion and the inner membrane fusion induced by mitofusins (Mfn1, Mfn2) and OPA1. Such a dynamic process is termed mitochondrial dynamics (Fig. 3).

Mitochondrial dynamics and mitophagy are two key processes to maintain mitochondrial homeostasis and normal physiological function. Ample evidence proved that inhibiting mitochondrial fission or enhancing mitochondrial fusion will weaken mitophagy [82, 83].

Many studies have shown that mitochondrial fission is a prerequisite for mitophagy [82, 84, 85]. Downregulation of Drp1 via transfection of Ad-shDrp1 can lead to a significant decrease in Lc3 protein in cardiomyocytes [85, 86]. Lc3 protein is a direct indicator of evaluating the degree of autophagy. Hence, insufficient autophagy due to downregulation of Lc3 leads to the accumulation of dysfunctional mitochondria in cardiomyocytes. BNIP3 is another mitophagy-related protein that induces autophagy. Overexpression of BNIP3 leads to an increase in Drp1-mediated mitochondrial division [84]. FUNDC1 can regulate mitochondrial fission by interacting with OPA1. Overexpression of FUNDC1 will promote mitochondrial fission and mitophagy, while knocking down FUNDC1 will induce mitochondrial fusion [72]. Mitochondrial fission and fusion counterbalance with each other under normal mitochondrial dynamic network. Some other studies have demonstrated that regulating mitochondrial fusion will have a similar effect on mitophagy like mitochondrial fission. For example, the reduction in fusion caused by OPA1 knockout also helps the elimination of dysfunctional mitochondria [87, 88]. However, conditional knockout of Mfn1/2 in mouse cardiomyocytes lead to an inversed result where dysfunctional mitochondria increased and cardiac hypertrophy happened due to impaired mitochondrial autophagy [89–91]. Thus, mitochondrial dynamics is an important part of mitophagy. However, mitochondrial dynamics also can regulate mitophagy.

### Mitophagy and mitochondrial biogenesis

Mitophagy and mitochondrial biogenesis are two interrelated and interactive processes, which have exactly the reverse effect in regulate mitochondrial number and content. Excessive mitophagy or elevated mitochondrial biogenesis will break up the delicate balance of mitochondrial homeostasis, inducing (mitophagic) cell death or necrosis eventually [92].

AMPK and CaMK are two compounds which can regulate mitophagy and directly activate PGC-1α to improve mitochondrial biogenesis. In addition, PGC-1α can also be stimulated increased NAD+ induced by AMPK. The effects of these proteins on PGC-1α are post-translational modifications. It has been reported that NAD+ can activate SIRT1 to deacetylates PGC-1α in response to the need of mitochondrial metabolism. As a bridge between mitophagy and mitochondrial biogenesis, AMPK also phosphorylates ULK1/2 to initiate the formation of autophagic vacuole. CaMK enzymes are also involved in mitophagy by stimulating AMPK when Ca2+ increased. Overexpression SIRT1 can stimulate the formation of autophagosomes [93]. Lysosomes act as final “incinerators stoker” to degrade mitochondria during mitochondrial clearance. It has been proved that PGC1a directly interacted with TFEB to enhance autophagy-lysosome pathway [94]. Moreover, there is a positive cycle between TFEB and PGC-1α interaction via CLEAR (coordinated lysosomal expression and regulation) motif to keep mitochondrial number and content in stable [94, 95]. CLEAR motif exists in mitochondrial genes of human or mouse, it also exists in other genes involved in autophagy [96]. It has been demonstrated that TFEB was a therapeutic target in kidney, muscle, heart diseases by regulating mitochondrial biogenesis via PGC-1α [97]. Moreover, PGC-1α activator (ZLN005) alleviated kidney injury by accelerating mitochondrial clearance in cisplatin-induced AKI mice, where the co-localization between LC3 and damaged mitochondria was increased [98, 99]. PGC-1α also show a protective effect against myocardial ischaemia-reperfusion injury, heart failure, but the drug for cardiac PGC-1α agonist is still immature [100, 101]. It has been reported that mice lacking cardiac PGC-1α were more easily developed heart failure than mice lacking systemic PGC-1α, but the reasons for this are unclear [100]. A breakthrough may come if mitophagy is taken into account.

The major mitophagic component-Parkin can also activate PGC-1α by inhibit Parkin-interacting substrate (PARIS), which is tended to bind to PGC-1α promotor to repress its expression in brain. Whether the system of Parkin-PARIS-regulated PGC-1α exists in heart need to be furtherly investigated. It is worth considering and studying whether PARIS is involved in the inconsistent levels of autophagy in different stage of ischemia reperfusion. Both mTOR and sirt1 are like balance weights on a scale, able to modify the regulators of mitophagy and mitochondrial biogenesis at either end of the scale in their own way. Proteins related with mitophagy (Atg families including LC3) or mitochondrial biogenesis (PGC-1α) can be deacetylated to induce mitophagy or mitochondrial biogenesis. But not all proteins interact with PGC-1α in a positive feedback loop as TFEB does. It’s well known that inhibiting mTOR will activate autophagy or mitophagy, but it has been reported that the action of mTOR will improve mitochondrial biogenesis accompanied with increased lysosomal biogenesis with repressed autophagy [102]. That means the regulation of mitophagy and mitochondrial biogenesis by mTOR occurs antagonistically.

## Mitophagy and cardiovascular disease

### Atherosclerosis

Atherosclerosis (AS) is, a chronic inflammatory disease of the large arteries initiated by lipid entry. In industrial countries, atherosclerosis is a primary disease among causes of heart disease and stroke. Endothelial adhesion molecules are highly expressed in arterial endothelial cells promoting differentiation of monocytes into macrophages, which subsequently turn into foam cells with lipid accumulation [103]. The pathogenesis of atherosclerosis is characterized by the accumulation of these macrophages, lipids, cholesterol, the migration and proliferation of vascular smooth muscle cells (VSMCs) [104]. Autophagy or mitophagy, as a cleaner, plays an indispensable role in removing this “accumulated waste” mentioned above. The role of autophagy or mitophagy does not have a single effect on these cells involved in atherosclerosis. Hypoactive autophagy will contribute to increased plaque formation in VSMCs, and increased ROS levels from damaged mitochondria in all cell types, on the contrary, hyperactive autophagy will generate autophagy-induced cytotoxicity in macrophage and VSMCs [105]. Atherosclerosis begins in the fetal aorta and slowly spreads to the coronary arteries and cerebral arteries in turn. During this complex process, plaque is formed [106].

Plaque is also termed fatty deposit, and its formation is related to matrix metabolism, calcification, and inflammation. Many scientists argue that plaques build up when the artery’s intima becomes damaged. Accumulated plaques narrow the arterial lumen and reduce blood flow, which leads to the occurrence and development of atherosclerosis [107]. Plaque rupture is the highest fatal cause in atherosclerosis, which is related with autophagy and mitophagy [108]. There were many articles indicated that it was insufficient autophagy and mitophagy that triggered inflammasome activation and eventually lead to the accumulation of plaque [109, 110]. Enhancing autophagy may help to delay cell death or ensure that the dysfunctional cells are efficiently cleared. Tacrolimus, as a mTOR inhibitor, helped to macrophage content in plaques, which was related with the unstable plaque [111]. Moreover, melatonin also shows a positive function in stabilizing atherosclerotic plaques by activating SIRT3/FOXO3a/Parkin-dependent mitophagy pathway and reducing inflammation [112]. Yu et al. put forward that autophagy and mitophagy was upregulated, on the contrary, mitochondrial respiration and mtDNA was reduced in human atherosclerotic plaques compared with normal arteries, which indicating impaired mitochondrial turnover [113]. In addition, Swaminathan et al. suggested that the autophagy marked by Lc3 was profoundly decreased in carotid plaques of symptomatic patient compared with asymptomatic patients [114]. The stabilization of plaque was weakened by impaired mitophagy. Their findings are not contradictory because of their different study background. The impaired mitochondrial renewal and turnover caused by imbalance between mitophagy and mitochondrial biogenesis will contribute to the ROS production.

ROS and inflammation play a vital role in the development of atherosclerosis. There is evidence that overproduction of ROS can damage mitochondrial DNA and lipid, which directly contributes to atherosclerosis and increase inflammation [115]. Excessive ROS also leads to endothelial dysfunction, with proliferation and apoptosis of VSMCs and macrophages, leading to atherosclerotic progression and possible plaque rupture. Worsely, excessive ROS will in turn damage other normal mitochondria, thus a negative cycle is created called ROS-induced ROS release (RIRR). Mitochondrial DNA damage with resultant mitochondrial dysfunction has been associated with the degree of atherosclerosis in early human atherosclerotic specimens and apoE^−/−^ mice with reduced LDL absorption from blood due to lacking apolipoprotein E [116].

Manganese superoxide dismutase (Mn-SOD) deficiency can accelerate mtDNA damage and enhance atherosclerosis phenotype in apoE^−/−^ mice [117]. Melatonin was found as a potential therapeutic drug to reduce inflammation and ROS by inhibiting JNK/Mff signaling and sustained mitochondrial homeostasis, thereby protecting endothelial cells against ox-LDL-induced damage [118].

Mitophagy and mitochondrial dynamics are cornerstones of mitochondrial quality control by removing damaged mitochondria to ensure normal mitochondrial function and cell homeostasis, which is disturbed in the pathogenesis of vascular diseases. The increased mitochondrial fragmentation and FIS1 expression have been found in venous endothelial cells of patients with type 2 diabetes [119]. What’s more, the expressions of FIS1 and Drp1 are also increased in human aortic endothelial cells cultured in high glucose medium. Fragmented mitochondria resulted from a 50% decrease of Mfn2 protein, also existed in synthetic VSMCs induced by platelet-derived growth factor-BB (PDGF) [120, 121]. Mitochondrial division inhibitor 1 (mdivi-1) is considered as an effective drug to protect against synthetic VSMCs by inhibiting mitochondria fission and attenuating cell proliferation [122]. On the other hand, Melatonin regulates mitophagy. It attenuates leukocyte hormone-1 β (IL-1β) secretion through SIRT3/FOXO3a/ Parkin-dependent signaling pathways to decrease the inflammatory factors secretion, preventing atherosclerotic plaques rupture [112]. Still, the protective effects were partially abolished by the autophagy inhibitor-3-MA. Atg7 deletion in VSMCs or mouse models has accelerated atherosclerosis development due to dysfunctional autophagy [123]. Defective autophagy in atherosclerosis is mainly due to dysfunctional lysosomes. Activating autophagy by elevating the autophagosomal marker Lc3-II can relieve asymptomatic patients with carotid plaques. Although enhancing autophagy and mitophagy show a protective effect on cardiac function, everolimus (mTOR inhibitor) is currently approved to reduce graft vasculopathy in heart transplant patients, not in atherosclerotic patients [124]. Interestingly, it has been reported that sirolimus and everolimus-eluting coronary artery stents show a protection in inhibition VSMCs proliferation [125]. We hope more analogs of rapamycin will be found to treat against atherosclerosis effectively and prevent coronary acute syndrome.

### Myocardial ischemia-reperfusion injury and myocardial infarction

Ischemic heart disease (IHD) is a major cause of death and disability worldwide, with a clinical manifestation of myocardial infarction and ischemic cardiomyopathy. During myocardial infarction and I/R, temporary or permanent occlusion of coronary arteries can cause multi-component consequences including insufficient oxygen and nutrient supply, intracellular acidification, mitochondrial Ca^2+^ overload, abnormal metabolism, the mitochondrial permeability transition pore complex (mPTP) opening. The mentioned above further resulted in mitochondrial dysfunction and increased ROS, ultimately leading to myocardial cell death and myocardial injury. Most of this myocardial damage is irreversible and it is difficult to achieve complete or partial recovery.

Mitochondrial function plays a decisive role in the sensitivity and recovery of I/R and MI injury. Excessive mitochondrial fission caused by I/R injury induces extrinsic apoptotic cell death; this process may contribute to the pathogenesis of postischemic cell death [126]. Inhibition of excessive mitochondrial fission is considered to be potential cardioprotection of I/R injury. For example, Drp1K38A shows a protective mitochondrial uncoupling effect against I/R in Drp1K38A-treated cardiomyocytes [127]. Myocardial infarct size was also significantly decreased in Drp1K38A-treated rats compared with controls. Mdivi-1 was considered as a beneficial drug for I/R injury by reducing infarct size, increasing the proportion of elongated interfibrillar mitochondria and delaying mPTP opening in the ischemic adult murine heart [128]. Another mitochondrial inhibitor, P110, has been shown to reduce myocardial infarction size during reperfusion and protect against poor left ventricular remodeling after myocardial infarction in adult rats [129]. Inhibiting Fis1-mediated fission in cardiomyocytes by miR-484 has also shown a reduced infarct size in a mouse myocardial infarction model [130].

**Fig. 4:**
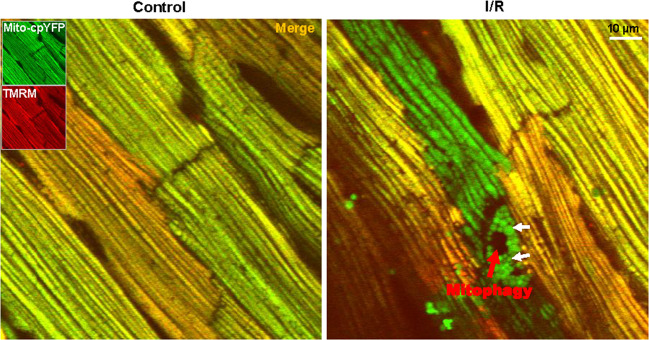
I/R induces autophagic degradation of mitochondria in cardiac. White arrow: Swollen mitochondria induced by the mPTP opening of the depolarized mitochondria that triggered by reperfusion after glucose deprivation. Red arrow: Depolarized mitochondria have been eliminated by mitophagy.

Autophagy plays a pivotal role in “housekeeping” for disposal of damaged or obsolete organelles such as mitochondria (mitophagy) and it is markedly upregulated during I/R. It should be pointed out that regulating autophagy may play a different role in different stages of I/R. For example, it has been suggested that the autophagy pathway may be protective by maintaining basal metabolic needs when the heart experiences an energy loss, such as ischemia, which is in contrast to reperfusion [131, 132]. In the ischemic heart, AMPK was activated to induce cardiomyocyte autophagy to remove malfunctioning mitochondria that otherwise lead to oxidative stress (Fig. 4). Both Park2^−/−^ mice and Pgam5^−/−^ mice show mitophagy inhibition after MI injury, which leads to increased heart infarction area, aggravated heart injury, and reduced cellular survival rate [2]. Unlike PINK1^−/−^ mice with increasingly susceptible to I/R, overexpression of PINK1 in cardiac cells protects against death by delaying the initiation of mPTP opening [4]. However, just one-sided seems to be the case since inhibition of autophagy actually alleviates heart injury in other situations. Inhibition of Beclin1 has been shown a protective effect for cardiomyocytes by preventing cell death in vivo [133]. What’s more, Becn1^+/−^ mice are more resistant to heart injury during reperfusion than WT mice. Similarly, Bnip3^−/−^ mice also show reduced myocardial injury and maintenance of cardiac function during ischemia/reperfusion [134]. Studies on Ulk1/Rab9/Rip1/Drp1 mitophagy mediated pathway have also shown a beneficial role in protecting the heart against ischemia by maintaining healthy mitochondria [135]. Autophagy was also induced by repetitive myocardial ischemia in chronically instrumented pigs, and cardiac function can also be restored after the coronary arteries return to normal flow, suggesting that autophagy may be vital for the survival of hibernating myocardia [136, 137].

It has been reported that the calcified aortic valve stenosis (CAVS) contributed to the myocardial infarction [138]. The trend of autophagy and mitophagy in CAVS was reverse in the articles of Deng et al. and Carracedo et al., which is because of their different compared group to the CAVS [139, 140]. Compared with the normal aortic valve referred by Deng et al., the autophagy level in CAVS was reduced, which was increased while compared with aortic regurgitation referred by Carracedo et al. The different result between them may indicate that the severity of the disease may have a different effect on autophagy. Marciano et al. confirmed this suppose in 2021. They argued that calcification aroused autophagic cell death, which was proved by the inconsistency between the decreased ATP production caused by impaired mitochondrial turnover and the enhancement of autophagy and mitophagy [141]. This result was also supported by Somers et al. in 2006, who revealed that it was autophagy in calcific aortic valve stenosis not apoptosis lead to cell death proved by immunohistochemical data [142]. Mitochondrial biogenesis also influences mitochondrial renewal and turnover. PGC-1α as the mitochondrial biogenesis regulator also has been proved to be downregulated in the failing heart [143].

### Cardiomyopathy

Cardiomyopathy (CM), is a group of myocardial diseases characterized by abnormal structure and function of the myocardium. Cardiovascular complications are the major risk factor of morbidity and mortality in T2DM. The main factors promoting the development of CM include hyperglycemia, insulin resistance of the whole body and heart, and the increase of free fatty acid (FFA) level, myocardial inflammation, oxidative stress, myocardial remodeling and fibrosis. In clinical common cardiomyopathy, autophagy and mitophagy have been frequently reported in the following kinds of diseases: dilated cardiomyopathy (DCM); hypertrophic cardiomyopathy (HCM), desmin-related cardiomyopathy (DRM) and restrictive cardiomyopathy (RCM). More and more evidence indicated that the alteration of autophagy and mitophagy is closely related to cardiomyopathy progression.

Beclin1 and Atg5 are two autophagy-related (Atg) proteins, however they show a different result between Becn1^+/−^ mice with alleviative myocardial remodeling and Atg5^+/−^ mice with aggravating myocardial hypertrophy in pathological myocardial remodeling [144, 145]. It’s known that Atg5 and Beclin1 both are related with autophagosome formation, but Beclin1 can induce autophagy independent of Atg5 indicating a complicated function in cardiomyopathy. It has been reported Beclin knockdown stimulated Rab-9 as a alternative autophagy and mitophagy with increased BNIP3, but Parkin was downregulated [146]. What’ more, mitochondrial biogenesis was also rescued by Beclin knockdown to relieve cardiomyopathy induced by high-fat diet. These results suggested that Beclin1 knockout did not inhibit autophagy and mitophagy, but instead activated other autophagy and BNIP3-mediated mitophagy, thus protecting heart function via enhance mitochondrial turnover. But the role of autophagy is not always an angel. It has been reported that decreased autophagy is an adaptive and protective result to protect heart function in type1 diabetes, while overexpressed Beclin1 contributed to aggravated cardiac injury [147]. Both genes are upstream of autophagy, Beclin and ATG5 knockout both reduced autophagy, but why were the results different? Unfortunately, the reason has not been found, and more research need to further explore. The pathobiology of DCM also exhibited partially heritability and reduced autophagy level. There were about 25% titin mutations and 6% Lamin A/C (LMNA) mutations in DCM [148]. Whether it was the increased formation of autophagosome or decreased (mitophagy) clearance contributed to Lc3 II increase has not been elaborated. The inhibition of cardiac autophagy or mitophagy in T1DM and T2DM may also lead to diabetic cardiomyopathy. Studies have shown that PINK1 and Parkin protein levels were significantly lower in T1DM hearts than in healthy hearts, consistent with reduced cardiac mitophagy [149].

Transferrin receptor (Tfrc) is responsible for iron uptake, which is important for cardiac function. Cardiac iron deficit by Tfrc deletion leads to severely ineffective mitophagy and develop lethal CM, indicating that mitophagy may be an effective therapeutic target for Tfrc-dependent CM [150]. Mitochondrial DNA (mtDNA) is generally degraded by DNase II in the mitophagy-lysosome system. Incomplete mtDNA degradation can stimulate cardiac inflammation, leading to heart failure. It has been proved that DNase II deficiency can lead to severe myocarditis and DCM, and result in premature death under pressure overload conditions [151]. Lc3 overexpression and enhancing Parkin-mediated mitophagy by inhibiting MST1 phosphorylation can protect against CM [152].

The autophagy level labeled by Lc3 significantly increased in HCM induced by Mybpc3 mutations and diabetic CM (type 2 diabetes). Rapamycin treated in Mybpc3 mice increased autophagy flux and reduced hypertrophy. It’s not just genetic mutations that can lead to cardiac hypertrophy, nurture or postnatal conditions are also included. It has been reported that the expression of PINK1 and Parkin was reduced in TAC-induced HCM, which indicating a downregulation of mitophagy. Moreover, it has been proved that loss of PINK1 lead to cardiac hypertrophy and mitochondrial dysfunction in mice at 2 months of age [153]. Xiong et al. demonstrated that accelerated PINK1-mediated mitophagy played a protective role in angiotensin-induced cardiac hypertrophy [154].

Tacrolimus treatment was also beneficial for attenuating myocardium damage and mitochondria function by preserving mitochondrial transmembrane potential [155]. However, Noda et al. has reported that tacrolimus triggered HCM in a patient with dermatomyositis [156]. The different result of tacrolimus treatment may be associated with inflammatory reaction and need to be further explored. Knocking out Parkin aggravated cardiac lipotoxicity and mitochondrial dysfunction under high-fat diet, which was reversed by Tat-Beclin1-activated mitophagy [157]. In addition, ALCAT1 show an abnormal increase in hypertrophic cardiomyopathy. Inhibiting ALCAT1 expression reduced ventricular fibrosis and mitigated HCM by increasing mitophagy level labeled with upregulation of PINK1, Lc3, and downregulation of p62 [158]. In conclusion, enhanced autophagy and mitophagy has shown a protective effect in cardiac hypertrophy, whether due to genetic mutations or other causes.

DRM is a kind of genetic cardiomyopathy caused by desmin- or αB-crystallin- mutations [159]. It has been reported that there was a significant increase of the desmin and insoluble CryAB levels in hearts of patients, which was also found in the end-stage ischemic HF [160]. In the early days, it was thought that the aberrant protein aggregation in DRM would inhibit autophagy, but in 2019, Pan et al. demonstrated that the level autophagy varies with TFEB during disease progression [161]. As the symptoms of the cardiac phenotypes become more apparent, the regulation of autophagy declined more and more, which changed from positive to negative when impaired TFEB signaling occurred as an inflection point. Knocking out Beclin1 further increasing interstitial fibrosis and accumulation of polyubiquitinated proteins in CryAB^R120G^ mice [162]. In contrast, accelerated autophagy via overexpressing Atg7, treating with SAHA, or overexpressing UBC9 has enhanced elimination of aggregated proteins in heart, and restored heart functions in CryAB^R120G^ mice [163–165]. Although mitochondria function was abnormal or the content of mitochondria is low in DRM, unfortunately, there are few studies on mitophagy in DRM [159]. Similar with DRM, dysfunctional autophagy and mitophagy has been associated with cardiomyopathy in the patient with a BAG3-Pro209Leu mutation and mice with abnormal BAG3 [166, 167].

### Myocardial hypertrophy and heart failure

Myocardial hypertrophy is a typical early adaptive response of the heart to increased pressure and mechanical stress. The direct cause of myocardial hypertrophy is the enlargement of cardiomyocyte size. Not all such increases in the cardiomyocyte area are harmful. Compensatory hypertrophy is a beneficial case in response to stress. The increased cardiomyocyte sarcomeres and cardiac mass were adaptive to normalize ventricular wall stress. However, chronic pressure will lead to cardiomyocyte cell death and irreversible damage to the heart and finally develop into Heart Failure (HF). The incidence of HF is related to age and it is as high as 20–30% in people aged 70–80 [168]. With age, the injured mitochondria cannot be cleared in time, caused by decreased autophagy and mitophagy in the heart, which leads to excessive ROS production and oxidative damage of various mitochondrial proteins.

Increased autophagy and mitophagy in multiple HF models was considered a protective response in cardiomyocytes [3]. Mitochondria-derived ROS activated AMPK, and then autophagy was activated. In addition, the cAMP/PKA and MAPK/ERK1/2 signaling pathways can also activate autophagy. Increased proteostasis caused by enhanced autophagy can alleviate cardiac hypertrophy and delay HF progression. Increased mitophagy is adaptive to the nutrient and energy requirements to pressure overload. Cytosolic p53 impairs mitophagy by binding with Parkin to disturb subsequent clearance of damaged mitochondria and further facilitates cardiac dysfunction and HF [169]. Mfn2 deficiency impeded Parkin-mediated mitophagy and contractility, which finally lead to myocardial hypertrophy and HF [170]. At the end-stage human HF, the expression level of PINK1 protein decreased significantly, indicating weakened mitophagy [171]. However, some scientists hold the opposite view, they argue that impaired mitophagy will contribute to negative pathological remodeling of myocardium. The activation of mitophagy leads to the transformation of the heart from adaptive compensatory hypertrophy to myocardial fibrosis, then develops into HF. There were only mild systolic dysfunction and no significant change in heart size of Becn1^+/−^ mice and cardiac-specific BECLIN 1 knockout mice. However, cardiac-specific overexpression of Beclin-1 resulted in converse effects [144]. Upregulated mitophagy under pressure overload aggravates myocardial hypertrophy and systolic dysfunction. In addition, cardiac-specific NIX knockout reduces myocardial fibrosis and apoptosis, and systolic dysfunction after TAC [172].

Over the years, many studies have suggested a link between heart failure and cancer sharing similar causes of disease, such as smoking and unhealthy diets. Researchers have shown that cardiac excreted factors such as serpinA3 and A1 during heart failure caused intestinal precancerous polyp growth [173]. Moreover, doxorubicin as an effective anti-cancer drug, has significant side effects in leading to cardiomyopathy and heart failure. Abdullah et al. reported that the cardiotoxicity of doxorubicin is mainly due to its inhibition of autophagolysosome degradation, accompanied by the backlog of damaged mitochondria and Lc3 [174]. This backlog of garbage, if not removed in time, can accelerate ROS generation and decrease ATP production by OXPHOS. A patient with dilated cardiomyopathy supported this conclusion, who show an excessive autophagy but abnormal lysosome function due to LAMP-2 lack [175].

Mitophagy is an essential factor for maintaining mitochondrial homeostasis. Insufficient mitochondrial clearance by mitophagy deficiency results in the accumulation of myocardial ROS, which triggers apoptosis. Lavandero et al. reported that the level of autophagy and Lc3 expression was decreased in patients with postoperative atrial fibrillation. However, excessive mitophagy leads to a decline in mitochondrial number and ATP production, leading to insufficient contraction of cardiomyocytes and HF deterioration. Although autophagy and mitophagy in HF are still controversial, they will be the new focus of HF therapy.

### “mPTP and mitophagy” in cardiac diseases

Long-lasting mPTP opening can cause mitochondrial dysfunction and cell death, which was closely related to the occurrence and development of various cardiac diseases. Understanding the link between mPTP and mitophagy in cardiac diseases may help to a better understanding of the mechanisms of cardiac disease. mPTP consist of a group of proteins that is responsible for transporting substances between the mitochondrial matrix and cytoplasmic. Currently recognized proteins include cyclophilin-D (CyP-D) located in matrix, voltage-dependent anion-selective channel 1 (VDAC1) located at OMM, the adenine nucleotide translocase (ANT) and the phosphate carrier (PiC) both located in inner membrane.

There are many similarities between mPTP opening and mitophagy occurrence, for example, the decrease of mitochondrial membrane potential, calcium overload, the change of ROS production. These similarities mean that there may be a certain connection between them. Excessive calcium can impair mitochondrial function and increase ROS, eventually triggering mitochondrial scavenging system. More seriously, calcium overload in clinic is often associated with arrhythmias and sudden death.

It has been demonstrated that a small portion of mPTP opening can initiate mitophagy to repair mitochondrial homeostasis via cleaning abnormal mitochondria, while excessive mPTP opening will induce mitophagy [176]. Both autophagy and mitophagy were increased in mouse hearts after myocardial infarction, while there was also accompanied by mPTP opening [177, 178]. It is not simple cause-and-effect relationship between mPTP opening and mitophagy, and the change between them do not always coincide. It has been shown that CyP-D knockout mice had a smaller myocardial infarct size, better preserved LV systolic function, and less mortality in post-myocardial infarction (MI) heart failure [179]. Moreover, other inhibitors of CyP-D also show a protective effect in post-MI heart failure. In addition, it has been proved that enhancing mitophagy provided a stronger protection against cardiac injury after acute MI [177]. In conclusion, either enhanced mitophagy or repressed mPTP opening can alleviate cardiac injury. Cyclosporine A, as a mPTPs inhibitor by binding with CyP-D, can also inhibit parkin recruitment when mitochondrial damaged [180]. The relationship between mPTP and mitophagy in MI injury needs to be further explored.

As the only ADP/ATP translocase in mitochondria, ANT also participate in mitophagy, which is independent of its role in ATP production and nucleotide exchange [181]. It has been demonstrated that ANT is required in pink/parkin-mediated mitophagy via stabilizing pink located in mitochondria [181]. Mice lacking ANT show an accumulation of abnormal mitochondria induced by blunted mitophagy indicating that ANT was important in keeping mitochondrial quality [181]. In addition, mice lacking ANT more easily developed concentric hypertrophy with dilation and had a higher oxidative phosphorylation level [182]. Clinically, patients with ANT1 deficiency often show cardiomyopathy, mitochondrial myopathy, lactic academia and other clinical cases [183]. Overexpressing myocardial ANT1 improved myocardial function and protected against hypertension-induced cardiac pathology with improved mitochondrial structure and function [184].

Repressing PiC expression protect against cardiac ischemic-reperfusion injury. The effect of PIC on autophagy is rarely reported [185]. Both ANT and PiC are important in ATP generation, which strongly supported cardiac work. It has been found that decreased ATP production and abnormal energetic metabolism in the failing heart [186].

It’s well-known that Vdac1 interacts with pro-apoptotic Bcl-2 members or anti-apoptotic Bcl-2 members, the former process is involved with the cytochrome C release, while the later inhibits VDAC1 oligomerization. Marked overexpression of VDAC1 has been found in post-myocardial infarction patients, as well as in patients with chronic ventricular dilatation\dysfunction, which means an important role of vdac1 [187]. The overexpressed VDAC1 will result in an excessive mPTP opening and the release of proapoptotic proteins including cytochrome C. It has been revealed that the loss of cytochrome C via vdac1 channel in mitochondria impaired and diminished mitophagy level [188]. The relationship between vdac1 and parkin-mediated mitophagy is still controversial. Some scientist thought that vdac1 was irrelevant to pink-parkin-mediated mitophagy. Others demonstrated that vdac1 is the critical substrate of parkin. According to the description by Jongkyeong Chung, there are two different ubiquitinated structures of VDAC1: VDAC1 monoubiquitination and VDAC1 polyubiquitination [189]. The former monoubiquitination inhibits apoptosis, the later polyubiquitination is crucial in parkin-mediated mitophagy. the absence of polyubiquitination impaired mitophagy and hindered parkin translocation to mitochondria. Parkin regulates mitophagy and apoptosis through different modifications of VDAC1. The mitophagy level is different in different stages of ischemia-reperfusion, but the mechanism is unknown, exploring vdac1 mono- or ploy-ubiquitination may help to better understand its role in mitophagy.

### Conclusion and perspectives

As essential organelles for eukaryotic cells, Mitochondria are involved in multiple cellular functions, including ATP production, free radical production, calcium homeostasis, and cell apoptosis. Thus, mitochondrial quality needs to be well maintained for the optimal performance of mitochondria. Mitophagy is an essential mitochondrial quality control mechanism that eliminates damaged mitochondria, coordinating with mitochondrial biogenesis to control mitochondrial homeostasis. Abnormal mitophagy is related to many cardiovascular diseases, including atherosclerosis, ischemia-reperfusion injury, cardiomyopathy, hypertrophy, and heart failure. Modulating mitophagy can be a target for relieving pathologies of illness. Mitochondria occupy ~40% of the volume of adult cardiomyocytes that could not proliferate. Each cardiomyocyte is valuable for the heart. Overregulation of mitophagy would cause cell death. How to control the optimal regulation of mitophagy? Maintaining the time and level of regulated mitophagy is possible using compounds.

Furthermore, mitophagy regulation can not completely restore mitochondrial homeostasis, a prerequisite for cellular functions. How to restore the homeostasis of dysfunctional mitochondria? The coordination between mitochondrial biogenesis and mitophagy has been destroyed under pathological conditions. The turnover of mitochondria could not efficiently work. Simultaneously regulating mitophagy and mitochondrial biogenesis may be necessary to restore mitochondrial homeostasis via promoting mitochondrial turnover.

## Supplementary information

Reproducibility checklist

## Data Availability

The data that supports the findings of this study is available from the corresponding author upon reasonable request.
